# Prevalence of pre‐eclampsia in 265 patients with an intracranial aneurysm, 393 female relatives versus a control cohort: A case–control study

**DOI:** 10.1111/ene.16113

**Published:** 2023-10-27

**Authors:** Satu Kotikoski, Juho Paavola, Heidi J. Nurmonen, Virve Kärkkäinen, Terhi J. Huuskonen, Jukka Huttunen, Timo Koivisto, Mikael von und zu Fraunberg, Juha E. Jääskeläinen, Antti E. Lindgren

**Affiliations:** ^1^ Neurosurgery of NeuroCenter, Kuopio University Hospital, Institute of Clinical Medicine University of Eastern Finland Kuopio Finland; ^2^ Department of Neurosurgery Oulu University Hospital Oulu Finland; ^3^ Research Unit of Clinical Medicine University of Oulu Oulu Finland; ^4^ Department of Clinical Radiology Kuopio University Hospital Kuopio Finland

**Keywords:** hypertension, intracranial aneurysm, pre‐eclampsia, stroke, subarachnoid hemorrhage

## Abstract

**Background and objectives:**

There is emerging evidence on the connection between pre‐eclampsia and saccular intracranial aneurysms (sIAs). Our aim was to study the prevalence of pre‐eclampsia in sIA patients, their female relatives, and matched controls, and to examine familial sIA disease and familial pre‐eclampsia in sIA patients' families.

**Methods:**

We included all female sIA patients in the Kuopio Intracranial Aneurysm Patient and Family Database from 1995 to 2018. First, we identified the sIA patients, their female relatives, and matched population controls with the first birth in 1987 or later and studied the prevalence of pre‐eclampsia. Second, all female sIA patients and all female relatives were analyzed for familial sIA disease and familial pre‐eclampsia. Using the Finnish nationwide health registries, we obtained data on drug purchases, hospital diagnoses, and causes of death.

**Results:**

In total, 265 sIA patients, 57 daughters, 167 sisters, 169 nieces, and 546 matched controls had the first birth in 1987 or later. Among them, 29 (11%) sIA patients, 5 (9%) daughters, 10 (6%) sisters, 10 (6%) nieces, and 32 (6%) controls had pre‐eclampsia. Of all the 1895 female sIA patients and 12,141 female relatives, 68 sIA patients and 375 relatives had pre‐eclampsia, including 32 families with familial pre‐eclampsia.

**Conclusions:**

Pre‐eclampsia was significantly more common in the sIA patients than in their matched controls. Familial sIA disease and familial pre‐eclampsia co‐occurred in seven families. Further studies of the mechanisms by which pre‐eclampsia could affect the walls of brain arteries and increase the rupture risk in sIA disease are indicated.

## INTRODUCTION

The prevalence of saccular intracranial aneurysms (sIAs), pouches on the bifurcations of intracranial extracerebral arteries, is around 3% in the general population [1]. Most sIAs remain undiscovered during life [2] if not incidentally found in neuroimaging or by screening for familial sIAs [3]. Aneurysmal subarachnoid hemorrhage (aSAH), caused by a rupture of the sIA wall, is the third most frequent form of stroke with high morbidity and mortality [4, 5, 6, 7, 8]. The risk factors for sIAs and aSAH include female sex, age, smoking, hypertension, and autosomal‐dominant polycystic kidney disease (ADPKD) [2, 9, 10]. sIA disease is a complex condition with at least 10% of sIA patients belonging to sIA families [11, 12].

About 4% of pregnancies in Europe are complicated by pre‐eclampsia [13], a severe multisystem disorder characterized by hypertension and maternal organ dysfunction. Globally, pre‐eclampsia causes substantial maternal mortality [14] and increased risks of hypertension and cardiovascular diseases [15, 16], type 2 diabetes [17], and stroke during pregnancy and later in life [16, 18]. ADPKD, a risk factor for sIA disease, is also a risk factor for pre‐eclampsia [19]. Pre‐eclampsia has a clear familial propensity, with both maternal and paternal factors increasing the risk [20], and familial linkage predicts more severe pre‐eclampsia [21]. Of all pre‐eclampsia cases, 20% are classified as severe [16, 22].

A possible connection between familial sIA disease and familial pre‐eclampsia has not been reported. In our previous study, pre‐eclampsia was more common in sIA patients than in their matched controls and sIA patients with pre‐eclampsia had more frequently irregularly‐shaped aneurysms [23]. The current study was conducted to verify the connection between pre‐eclampsia and sIA disease in an extended study population. Additionally, we included female relatives to study the familial linkage of pre‐eclampsia and sIAs. In the present study, we analyzed the prevalence of pre‐eclampsia in 265 sIA females with the first birth since 1987, their 393 female relatives, and 546 matched population controls, using data in the Kuopio Intracranial Aneurysm (IA) Patient and Family Database and data from the Finnish nationwide health registries [24, 25, 26]. Additionally, we examined all sIA females with the first sIA disease diagnosis from 1995 to 2018 (*n* = 1895) and all female relatives (*n* = 12,141) for the prevalence of familial sIA disease and familial pre‐eclampsia. We hypothesized about a familial aggregation of sIA disease and pre‐eclampsia.

## METHODS

### Catchment population of Kuopio University Hospital

Kuopio University Hospital (KUH) served a defined catchment population of about 850,000 in Eastern Finland during the study period from 1995 to 2018. All patients with verified aSAH by computed tomography (CT) or spinal tap were admitted to KUH for angiography and treatment if not moribund or very aged. Unruptured sIAs were diagnosed by four‐vessel digital subtraction angiography, magnetic resonance angiography, or CT angiography.

### Kuopio IA Patient and Family Database

KUH Neurosurgery maintains a database of all patients with ruptured and unruptured intracranial aneurysms admitted to KUH since 1980 and has been prospective since 1990 [11]. A full‐time database manager operates the database, interviews all new patients and follow‐up visits, and codes this information into variables, including sIA family history defined as at least two (≥2) affected first‐degree relatives [11]. Data for prescribed drug purchases, hospital diagnoses, and causes of death were obtained from national registries using Finnish personal identification codes, fused into the database, and analyzed [2, 27].

The sIA patients' first‐degree relatives (parents, children, siblings), nieces, and nephews were identified using Finnish personal identification codes. Random controls (three for each sIA patient in the Kuopio IA Database) were selected by the Digital and Population Data Services Agency and matched by age, sex, and birthplace, representing the general population. The date of the first sIA‐related admission was the index date for matching, at the time all controls were alive. Relatives' and controls' data for prescribed drug purchases, hospital diagnoses, and causes of death were obtained from the national registries using Finnish personal identification codes, fused into the database, and analyzed.

### Definitions of familial sIA disease and familial pre‐eclampsia

In the present study, familial sIA disease was defined as ≥2 affected first‐degree relatives in the same family [12, 25, 28]. Familial pre‐eclampsia was defined as ≥2 affected first‐degree relatives in the same family. The sIA + pre‐eclampsia families had ≥2 first‐degree relatives with sIA disease and ≥2 first‐degree relatives with pre‐eclampsia.

### Study population of 265 sIA patients, 393 female relatives, and 546 controls

**FIGURE 1:**
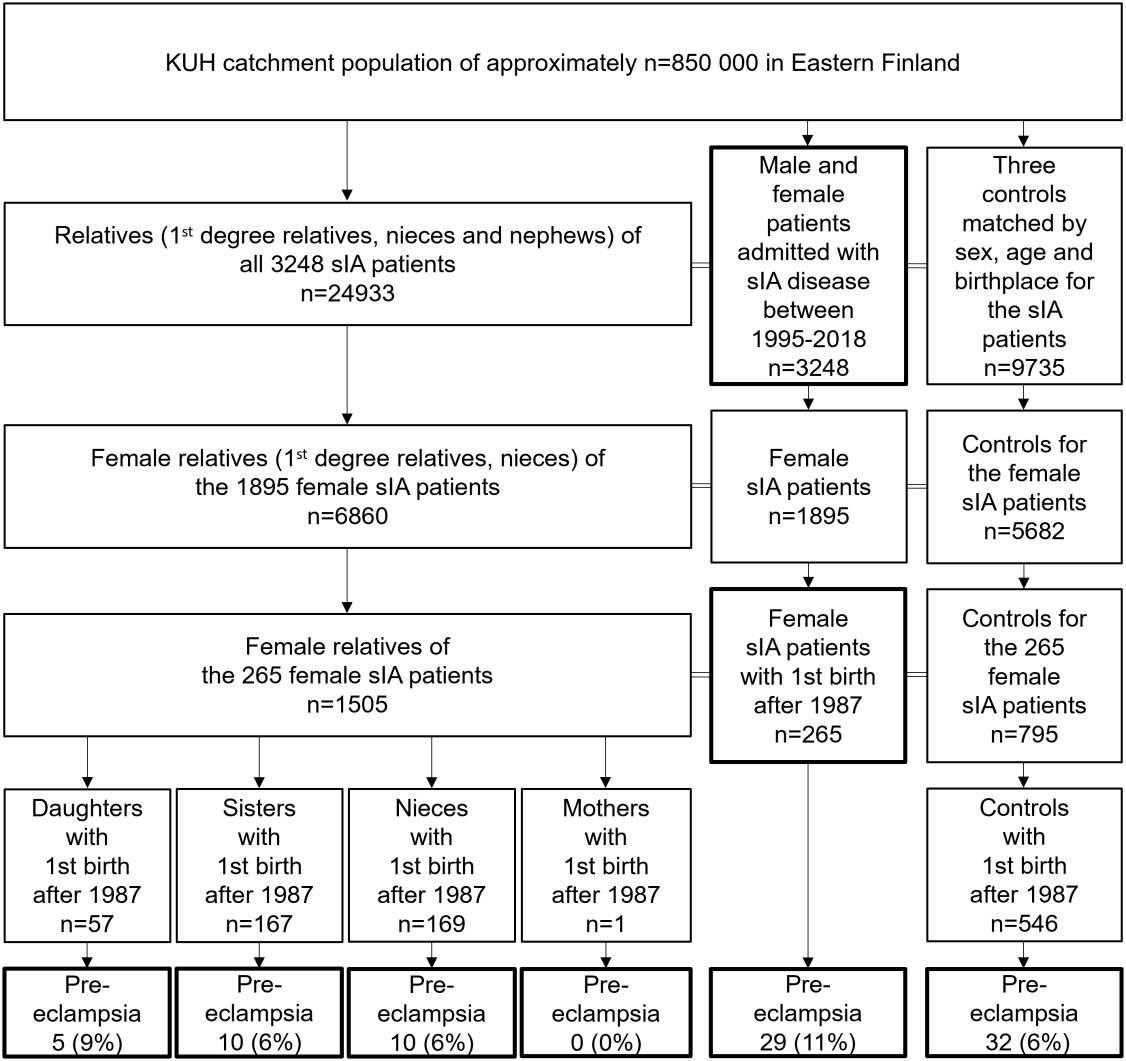
Flowchart for the 1895 female patients with the first aneurysmal subarachnoid hemorrhage (aSAH) or an unruptured saccular intracranial aneurysm (sIA) admitted to the Kuopio University Hospital (KUH) from the Eastern Finnish catchment population from 1995 to 2018. Data for the sIA patients' relatives as well as the data for population controls were obtained from the Digital and Population Data Services Agency. For each patient, three random female controls were matched by age and birthplace. Clinical data for each patient and matched control were obtained from the Finnish national registries. A total of 265 female sIA patients, 394 female relatives, and 546 controls, who had first given birth in 1987 or later, were identified, and their pre‐eclampsia diagnoses searched. Mothers were excluded due to insufficient data, resulting in 393 female relatives in the final study population.

The basic study population consisted of 1895 sIA females (Figure 1) who met the following criteria:

1. A citizen of Finland and resident of the KUH catchment area at the first diagnosis of sIA disease between January 1, 1995 and December 31, 2018.
2. Verification of sIA disease with four‐vessel angiography.
3. Patients with other types of IAs (fusiform, traumatic, mycotic, unknown) excluded.
4. The end of the follow‐up at death or December 31, 2019.

First, we searched sIA patients who, additionally, met the criterion:

5 The first birth in 1987 or later in Finland, the first year of comprehensive pregnancy and birth data for all patients, relatives, and controls available. We identified 265 sIA females who met the criterion. Of their 1505 female relatives (first‐degree relatives, nieces), 394 first gave birth in 1987 or later. Only one mother first gave birth in 1987 or later; therefore, mothers were excluded, resulting in 393 female relatives in the final study population. We identified 546 matched population controls for the 265 sIA patients matched by: age, sex, birthplace, alive at the index date, and first birth in 1987 or later (Figure 1).

### Study population of 1895 female sIA patients and 12,141 female relatives

Additionally, we searched the basic study population for all sIA females (*n* = 1895), and all female relatives (*n* = 12,141), who met the criterion:

6 Pre‐eclampsia diagnosis in any pregnancy.

**FIGURE 2:**
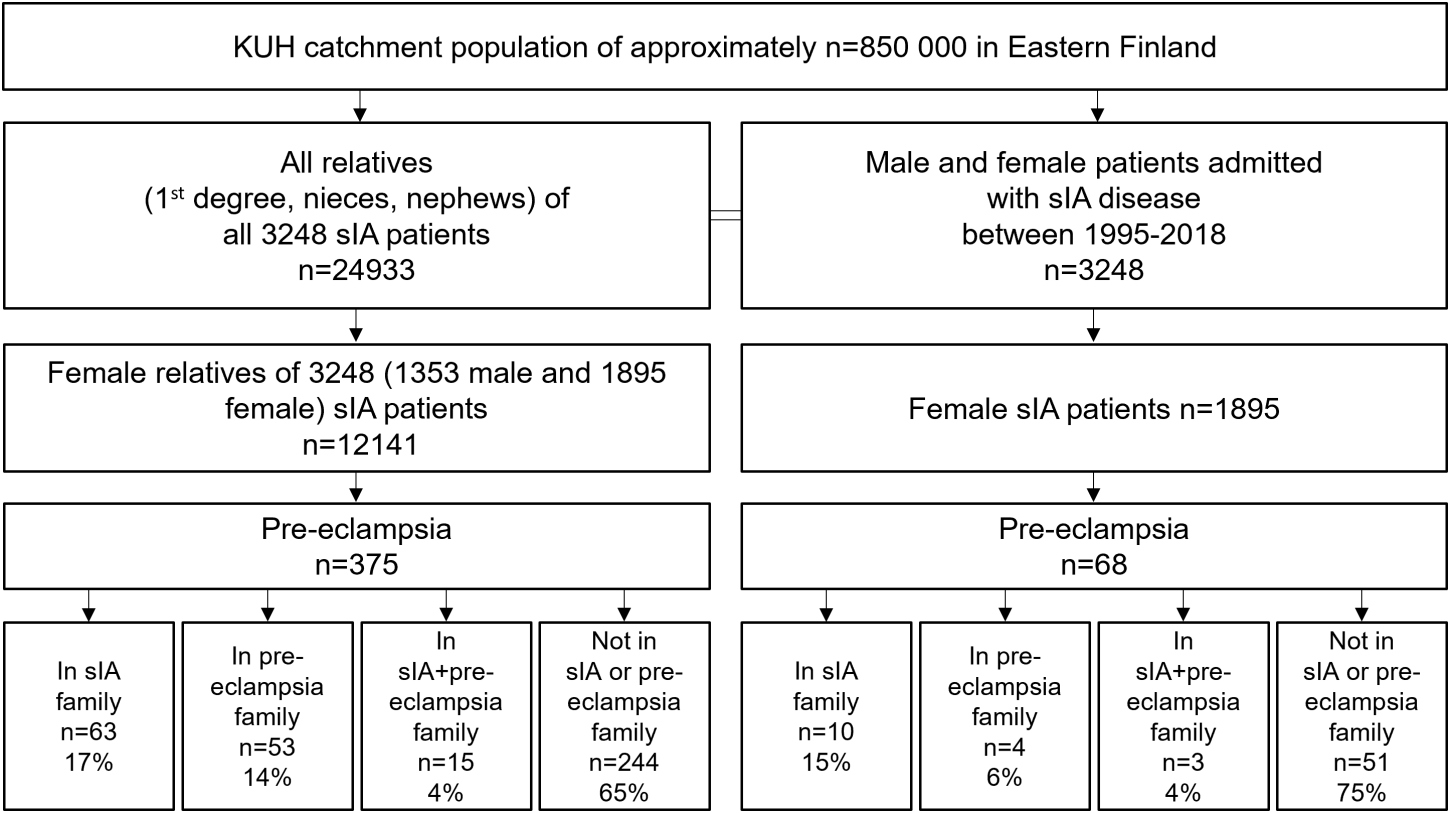
Flowchart for the search for all female saccular intracranial aneurysm (sIA) patients and all female relatives with ICD‐8, ICD‐9, or ICD‐10 pre‐eclampsia diagnosis. A total of 68 sIA females and 375 female relatives with pre‐eclampsia were identified. ICD, International Statistical Classification of Diseases and Related Health Problems; KUH, Kuopio University Hospital.

We identified 68 sIA patients and 375 female relatives who met this criterion. We included both sIA females' and sIA males' female relatives (Figure 2).

### Hospital diagnoses (1969–2019) and causes of death (1971–2018)

We used the International Statistical Classification of Diseases and Related Health Problems (ICD) codes from revisions 8, 9, and 10; in Finland ICD‐8 was used between 1969 and 1986, ICD‐9 was used between 1987 and 1995, and ICD‐10 since 1996, obtained from the Finnish national registries (Table S1). Hospital‐based ICD diagnoses were acquired from the Care Register of the Finnish National Institute for Health and Welfare (“HILMO”). Causes of death were acquired from Statistics Finland. Relatives who were additionally sIA patients in the Kuopio Intracranial Aneurysm Patient and Family Database were also included in relatives with IA disease. Superimposed pre‐eclampsia and eclampsia were included in pre‐eclampsia. In 2013, the American College of Obstetricians published new guidelines for diagnosing pre‐eclampsia, where the presence of proteinuria is no longer required if other signs of maternal organ dysfunction are present [29]. Previously, the diagnostic criteria for pre‐eclampsia in Finland were a new‐onset hypertension after the 20th gestational week combined with proteinuria ≥300 mg per day.

### Drug purchase data (1994–2019)

The drug purchase data, obtained from the Social Insurance Institution of Finland using Finnish personal identification codes, contained information since the first purchase date and the number of purchases until the last date. Drug use was determined as ≥2 purchases of prescribed drugs at any time during the study period [30]. Drugs were classified according to the Anatomical Therapeutic Chemical Classification maintained by the World Health Organization. Drug‐treated hypertension was defined as the purchase of prescribed anti‐hypertensive drugs: C02 (antihypertensives); C03 (diuretics; thiazides); C04 (peripheral vasodilators); C07 (beta‐blocking agents); C08 (calcium channel blockers); C09 (agents acting on the renin–angiotensin system). Drug‐treated type 2 diabetes was defined as the purchase of prescribed A10B blood glucose‐lowering drugs.

### Literature review

A PubMed search for articles in the English language on humans between 1990 and 2022 with the search terms (preeclampsia or pre‐eclampsia) AND (stroke or “intracerebral haemorrhage” or “intracerebral hemorrhage” or “subarachnoid haemorrhage” or “subarachnoid hemorrhage” or SAH or aneurysm*) resulted in 727 hits. No relevant cohorts were identified, except for our previous study, which to our knowledge is the first study on pre‐eclampsia among sIA patients [23].

### Statistical analysis

Distribution of variables was expressed in medians and interquartile ranges for the continuous variables and proportions for the categorical variables. In group comparisons the chi‐square or Fisher's exact test was used for the categorical variables as appropriate. *P* values <0.05 were considered significant. IBM SPSS Statistics 26.0 was used. There were no missing values.

### Standard protocol approvals, registrations, and patient consents

This research was authorized by the Research Ethics Committee of the KUH. Written consent was obtained from all patients before their data were added to the Kuopio IA Database. The need for additional control or relative consent for this registry study was waived by the Ethics Committee of KUH, as no study participants were contacted. Patient data integration from the nationwide registries was completed with the endorsement of the Ministry of Social Affairs and Health of Finland.

## RESULTS

### Study cohort of 265 sIA patients

We identified 265 sIA females between 1995 and 2018 with the first birth since 1987 (Figure 1 and Table 1). The 265 sIA patients gave birth to 602 (median, 2) children. Familial sIA disease was more frequent with unruptured sIAs than with aSAH (35% vs. 17%), likely due to sIA family screening.

**TABLE 1:** Characteristics of the study population of 265 female patients with saccular intracranial aneurysm disease or first aneurysmal subarachnoid hemorrhage admitted to the Kuopio University Hospital from the Eastern Finnish catchment population from 1995 to 2018, their 393 female relatives, and 546 matched female population controls with particular reference to the occurrence of pre‐eclampsia, clinical data of pregnancies, and registered drug use.

Females with first birth in 1987 or later						
Characteristic	Females with unruptured sIA (*n* = 127)	Females with aSAH (*n* = 138)	Daughters (*n* = 57)	Sisters (*n* = 167)	Nieces (*n* = 169)	Controls (*n* = 546)
Median age at sIA diagnosis (years) (quartiles)	45 (37–50)	44 (36–49)				
Familial sIA disease	45 (35%)	23 (17%)				
Multiple sIAs	26 (20%)	38 (28%)				
ADPKD	1 (1%)	3 (2%)	0 (0%)	2 (1%)	0 (0%)	1 (0%)
Median age at the end of follow‐up (years) (quartiles)	53 (45–58)	53 (47–56)	28 (25–30)	49 (40–53)	34 (30–40)	53 (47–56)
Pre‐eclampsia	12 (9%)	17 (12%)	5 (9%)	10 (6%)	10 (6%)	32 (6%)
Median age at first birth (years) (quartiles)	28 (24–32)	27 (24–30)	24 (22–26)	27 (23–31)	25 (22–28)	28 (25–31)
Median age at first pre‐eclampsia (years) (quartiles)	32 (28–36)	33 (24–38)	25 (24–26)	30 (28–34)	21 (20–29)	30 (25–33)
Severe pre‐eclampsia	2/12 (17%)	5/17 (29%)	0/5 (0%)	2/10 (20%)	2/10 (20%)	11/32 (34%)
Gestational diabetes	16 (13%)	14 (10%)	8 (14%)	20 (12%)	32 (19%)	67 (12%)
Use of antihypertensive drugs	74 (58%)	89 (64%)	5 (9%)	69 (41%)	31 (18%)	188 (34%)
Use of type 2 diabetes drugs	11 (9%)	6 (4%)	0 (0%)	8 (5%)	7 (4%)	37 (7%)

Abbreviations: ADPKD, autosomal‐dominant polycystic kidney disease; aSAH, aneurysmal subarachnoid hemorrhage; sIA, saccular intracranial aneurysm.

### Pre‐eclampsia in 265 sIA patients

Of the 265 sIA patients, 29 (11%) had pre‐eclampsia (Figure 1 and Table 1), including 17/138 (12%) aSAH patients. Among 29 pre‐eclampsia patients, 25/29 had the sIA diagnosis after the first pre‐eclamptic pregnancy, and the pre‐eclampsia diagnosis preceded the sIA diagnosis by a median of 13 years (quartiles 7–18). Pre‐eclampsia reoccurred in 1/12 (8%) of the patients with unruptured sIAs and 3/17 (18%) of the aSAH patients with pre‐eclampsia. Severe pre‐eclampsia was more common in patients with aSAH (5/17, 29%) than with unruptured sIAs (2/12, 17%). Familial sIA disease was more common in the aSAH patients with pre‐eclampsia (4/17, 24%) than without pre‐eclampsia (19/121, 16%). ADPKD was more frequent in the sIA patients with pre‐eclampsia (2/29, 7%) than without pre‐eclampsia (2/236, 1%). Among sIA patients with pre‐eclampsia, having a first‐degree relative with pre‐eclampsia was more common with aSAH than with unruptured sIAs (18% vs. 0%).

### Pre‐eclampsia in 393 female relatives and 546 controls

The frequency of pre‐eclampsia was 5/57 (9%) in daughters, 10/167 (6%) in sisters, and 10/169 (6%) in nieces. A total of 32 (6%) controls had pre‐eclampsia, considerably fewer than the sIA patients (*p* = 0.01), with an increased risk of pre‐eclampsia for the sIA patients compared with controls with odds ratio 1.97 (95% confidence interval, 1.17–3.34) (Table 1). When only unruptured sIA patients were compared with the controls, no statistically significant difference was reached (*p* = 0.14). There was no statistically significant difference in the frequency of pre‐eclampsia in the relatives and the controls.

### Use of antihypertensive drugs in 265 sIA patients, 393 female relatives, and 546 controls

Among 265 sIA females, 163 (62%) used antihypertensive drugs (Table 1). Of the 29 sIA patients with pre‐eclampsia, 19 (66%) used antihypertensive drugs compared with 144/236 (61%) sIA patients without pre‐eclampsia. Of the 32 matched controls with pre‐eclampsia, 16 (50%) used antihypertensive drugs compared with the 172 (33%) controls without pre‐eclampsia. A total of 9/25 (36%) female relatives with pre‐eclampsia used antihypertensive drugs in contrast to 96/368 (26%) female relatives without pre‐eclampsia. With severe pre‐eclampsia, antihypertensive drug use was more common in the controls (8/11 = 73%) than in the sIA patients (4/7 = 57%).

### Comparison of the 29 sIA patients with pre‐eclampsia and their 179 female relatives with the 236 sIA females without pre‐eclampsia and their 1326 female relatives

We analyzed comorbidities in the study population of 265 sIA patients and all their 1505 female relatives (Figure 1). There were no significant differences in the frequencies of IA disease, intracerebral hemorrhage, ischemic stroke, ADPKD diagnoses, in the use of antihypertensive or type 2 diabetes drugs, or in mortality when comparing the 179 female relatives of the 29 sIA females with pre‐eclampsia and the 1326 female relatives of the 236 sIA females without pre‐eclampsia. The frequency of pre‐eclampsia was higher in the 36 sisters of the 29 sIA patients with pre‐eclampsia compared with the 306 sisters of the 236 sIA females without pre‐eclampsia (8% vs. 4%), with no noticeable prevalence difference in the other relatives.

### Familial sIA disease and familial pre‐eclampsia in 68 sIA patients and 375 female relatives with pre‐eclampsia

In addition to the basic study population of 265 sIA patients, 393 female relatives, and 546 controls (Figure 1), we analyzed all 1895 sIA females and all 12,141 female relatives for pre‐eclampsia in the Kuopio IA Database between 1995 and 2018 (Figure 2). Among 68 sIA patients with pre‐eclampsia, 40 (59%) had aSAH. Of the 375 female relatives with pre‐eclampsia (111 daughters, 115 sisters, 13 mothers, 136 nieces), 201 (54%) were relatives to sIA females and 174 (46%) to sIA males. Of 68 sIA patients and 375 female relatives with pre‐eclampsia, 75 (17%) had first‐degree relative(s) with pre‐eclampsia and 46 (10%) had second‐degree relative(s) with pre‐eclampsia, distributed similarly in the sIA females' and sIA males' relatives.

**FIGURE 3:**
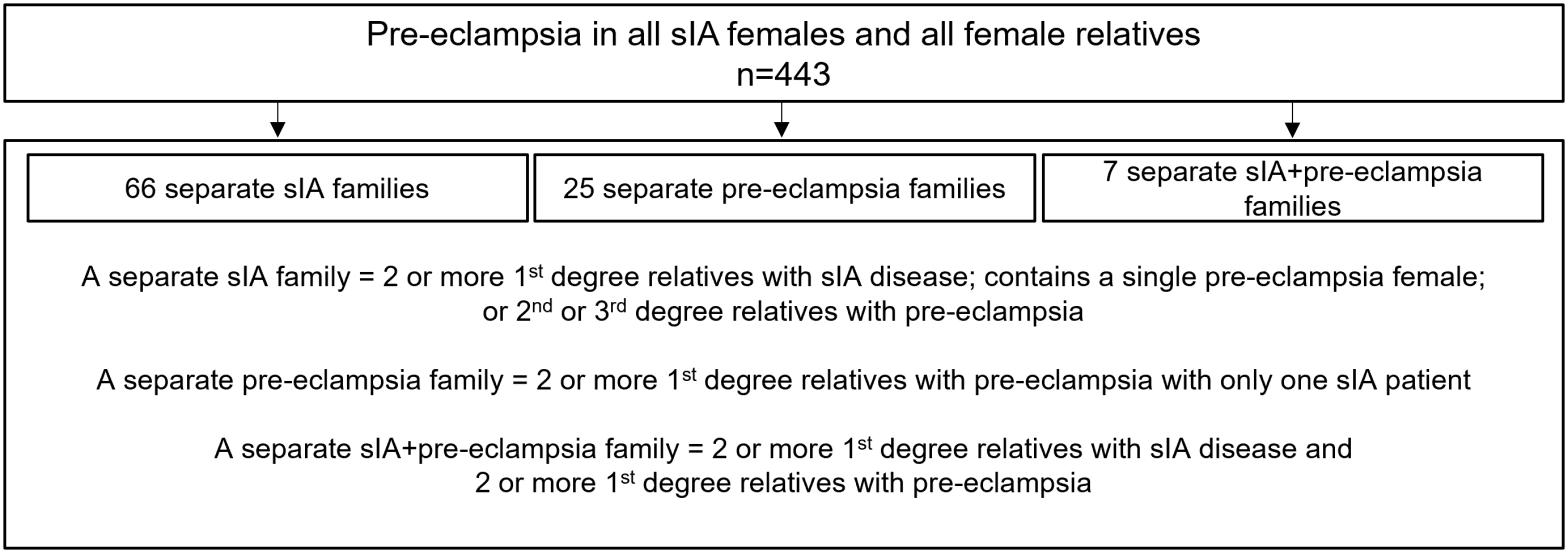
Identification of all separate saccular intracranial aneurysm (sIA) families, pre‐eclampsia families, and sIA + pre‐eclampsia families among 68 sIA patients with pre‐eclampsia and 375 female relatives with pre‐eclampsia.

We identified in total 32 families with familial pre‐eclampsia (Figure 3); 20 were sIA females' families and 12 sIA males' families. We found seven sIA + pre‐eclampsia families; 6/7 were sIA females' families. There were no ADPKD diagnoses in the 32 pre‐eclampsia families.

Severe pre‐eclampsia was more common with the familial sIA disease (6/13, 46%) than without the familial sIA disease (11/55, 20%) in the 68 sIA patients (*p* = 0.057). Among 68 sIA patients and 375 female relatives with pre‐eclampsia, the use of antihypertensive drugs did not significantly differ between the studied groups: sIA family (63%), pre‐eclampsia family (51%), sIA + pre‐eclampsia family (61%), and not in sIA or pre‐eclampsia family (53%) (Figure 2). There was no difference in antihypertensive drug use between unruptured (22/28, 79%) and ruptured (30/40, 75%) sIA patients.

Of the 375 female relatives with pre‐eclampsia, 3% (3/111) of the daughters, 5% (6/115) of the sisters, 8% (1/13) of the mothers, and none of the nieces had an IA disease diagnosis and 10% (11/115) of the sisters with pre‐eclampsia had an ischemic stroke diagnosis, more often than other female relatives with pre‐eclampsia.

## DISCUSSION

In our study population of 265 sIA females with the first birth since 1987, 11% had pre‐eclampsia compared with 6% of matched controls. Severe pre‐eclampsia was more common (29% vs. 17%) and pre‐eclampsia recurred more often (18% vs. 8%) with aSAH than with unruptured sIAs, respectively. We identified 32 families with familial pre‐eclampsia, and familial sIA disease co‐occurred in seven (22%) of the families. Severe pre‐eclampsia was more common with familial sIA disease than without familial sIA disease (46% vs. 20%) among sIA patients. Collectively, our results add to the accumulating evidence of the association between pre‐eclampsia and sIA disease.

Pre‐eclampsia was significantly more frequent in the 265 patients with sIA than in their 546 population controls, in line with our previous study [23]. In addition, irregularly‐shaped aneurysms were more frequent in the sIA patients with pre‐eclampsia in our previous study, a feature associated with an elevated risk of sIA wall rupture [23, 31]. The risk for sIA disease and aSAH in patients with a history of pre‐eclampsia has remained unclear, as earlier studies have primarily inspected stroke in general [16], long‐term risk of intracerebral hemorrhage [32], and pregnancy‐related SAH [33], as opposed to sIA disease.

The pathophysiology of pre‐eclampsia is not entirely understood; the prevailing theory is that although early‐ and late‐onset pre‐eclampsia share some risk factors, they could be different entities [34]. Early‐onset disease is characterized by defective placentation, whereas late‐onset disease is often influenced by maternal cardiovascular and metabolic risk factors [35]. Early‐onset pre‐eclampsia has the strongest genetic predisposition [36] and is often severe [22]. Both maternal and paternal familial linkages have been found; for instance, higher rates of pre‐eclampsia in pregnancies fathered by men who were born from pre‐eclamptic pregnancies [20, 21]. Genome‐wide linkage scans of pre‐eclampsia have identified significant loci in 2p13 [37], 2p25 [38], and 9p13 [38]. Genome‐wide association studies (GWAS) of pre‐eclampsia have identified several risk loci associated with hypertension [39, 40]. Of the 17 risk loci for intracranial aneurysms in the largest international GWAS [41], rs2681472 has been associated with pre‐eclampsia [42] and, additionally, with blood pressure, cardiovascular, and coronary artery disease [41]. Of the potential causative genes in the aforementioned GWAS of intracranial aneurysms, SLC22A4/OCTN1 and SLC22A5/OCTN2 have been linked to pre‐eclampsia [43, 44, 45].

Pre‐existing hypertension and ADPKD increase the risk of both pre‐eclampsia [19, 46] and sIA disease [2, 10]. In our study, antihypertensive drug use was similar in the 265 sIA patients with and without pre‐eclampsia (66% vs. 61%). When inspecting all sIA patients with pre‐eclampsia (*n* = 68), there was no difference in antihypertensive drug use in patients with unruptured and ruptured sIAs (79% vs. 75%). These findings suggest that hypertension does not solely explain the increased prevalence of pre‐eclampsia or rupture risk among sIA females. ADPKD was more common with than without pre‐eclampsia among 265 sIA patients (7% vs. 1%), but not in the female relatives or in the controls. There were no ADPKD diagnoses in 32 pre‐eclampsia families, thus ADPKD does not explain the co‐occurrence of sIA disease and pre‐eclampsia in these families. In our basic study population, severe pre‐eclampsia was more common with aSAH (29%) than with unruptured sIA disease (17%). However, severe pre‐eclampsia was also frequent (34%) in controls. Interestingly, in controls with severe pre‐eclampsia, antihypertensive drug use was more frequent than in the sIA patients with severe pre‐eclampsia (73% vs. 57%). This could illustrate different etiological features, although no apparent distinctions were observed in the studied variables. Nevertheless, hypertension is frequently documented after severe pre‐eclampsia [47].

Both pre‐eclampsia and sIA disease are complex conditions with clear familial components [12, 36]. The prevalence of pre‐eclampsia in the female relatives did not differ from the controls, indicating that familial aggregation of pre‐eclampsia does not explain the higher prevalence in sIA females. A slightly higher prevalence in daughters (9%) might originate from behavioral or methodological factors, as maternal obesity in Finland has increased rapidly [48], and the new guidelines for diagnosing pre‐eclampsia were published in 2013 [29]. We identified seven families with both familial sIA disease and familial pre‐eclampsia, fewer than initially expected. Prevalence of severe pre‐eclampsia was highest in the sIA + pre‐eclampsia‐families, while antihypertensive drug use did not notably differ from other sIA patients and relatives with pre‐eclampsia. In previous studies, hypertension has not been associated with familial sIA disease [2, 49]. It seems that familial sIA disease and familial pre‐eclampsia do not significantly share risk factors. However, both diseases may result in more severe forms when they co‐occur.

Our study has strengths originating from the Nordic health care system. Finland is divided into separate catchment areas for the KUH and the four other university hospitals, allowing the formation of unselected and minimally biased disease cohorts. The Finnish personal identification code system enables the congregation of accurate population and clinical data registries, with few individuals lost to follow‐up. The present study included only patients with four‐vessel angiography‐verified sIAs, excluding other types of IAs. All antihypertensive and type 2 diabetes drugs are sold only by physicians' prescriptions in the pharmacies in Finland, and all drug purchases were traced from the national registry for this study. In Finland, maternity and child health clinics monitor pregnancies with standard follow‐up visits with regular blood pressure level screenings. In Finland, nearly all births occur in hospitals (99.4% in 2020) [50] ensuring comprehensive national pregnancy and birth data available for the study period, and therefore our pre‐eclampsia data are minimally biased.

This study also has limitations. Our study was retrospective while the database was prospective during the study period. One weakness is the limited size of our cohort. We chose to use the ICD‐8, ICD‐9, and ICD‐10‐codes to categorize sIA patients, relatives, and controls into individuals with and without pre‐eclampsia. We did not have access to the ICD‐8 codes for patients with the first sIA diagnosis between 2015 and 2018, including those patients' relatives and controls, which might have led to undiscovered pre‐eclampsia cases. There were no data available for the relatives of the controls. Because of the age distribution of our study population, there are insufficient pregnancy data for the sIA patients' mothers, daughters, and nieces. Unfortunately, the sIA registry does not maintain reliable data on body mass index or smoking.

Among the first‐degree relatives, no distinction was made between full or half‐siblings, which might have led to underestimated sibling influence. In studying the familial aggregation of pre‐eclampsia we chose to include all sIA females' and sIA males' female relatives, considering maternal and paternal linkages in pre‐eclampsia [21]. We did not have data for the sIA males' partners, which apparently resulted in undiscovered pre‐eclampsia families.

Considering clinical practice, the history of pre‐eclampsia indicates an elevated risk of sIA disease and may associate with an elevated risk for instability in the yet unruptured sIA walls. Confirmation of this result in other population‐based datasets is warranted. Further studies of the mechanisms by which pre‐eclampsia could affect the walls of brain arteries and increase the risk of aneurysm rupture are indicated.

## AUTHOR CONTRIBUTIONS

**Satu Kotikoski:** Investigation; writing – original draft; visualization; formal analysis; data curation; funding acquisition. **Juho Paavola:** Data curation; writing – review and editing; investigation. **Heidi J. Nurmonen:** Writing – review and editing; investigation; data curation. **Virve Kärkkäinen:** Funding acquisition; writing – review and editing; project administration. **Jukka Huttunen:** Writing – review and editing; methodology; investigation; data curation. **Timo Koivisto:** Writing – review and editing; resources; data curation. **Mikael von und zu Fraunberg:** Supervision; writing – review and editing; data curation; validation.

## FUNDING INFORMATION

This study was funded by Kuopio University Hospital, Maire Taponen Foundation, The Finnish Medical Foundation, the North Savo Regional Fund of Finnish Cultural Foundation, Petri Honkanen Foundation, Pro Humanitate Foundation, Päivikki and Sakari Sohlberg Foundation, and the Academy of Finland.

## CONFLICT OF INTEREST STATEMENT

The authors have stated explicitly that there are no conflicts of interest in connection with this article.

## Supporting information


Table S1.

## Data Availability

The data that support the findings of this study are available on request from the corresponding author. The data are not publicly available due to privacy or ethical restrictions.
