# Supplementary material for: Prevalence of pre‐eclampsia in 265 patients with an intracranial aneurysm, 393 female relatives versus a control cohort: A case–control study
Source: Eur J Neurol. 2023 Oct 27;31(2):e16113. doi: 10.1111/ene.16113 (PMC11235792; doi:10.1111/ene.16113)
Supplement: Supplementary file 1 — Table S1. [file ENE-31-e16113-s001.docx]

Supplementary Materials

eTable 1. Hospital diagnoses obtained from the Finnish national registries; ICD-8, ICD-9, and ICD-10 codes for the sIA patients, their relatives, and matched population controls.

| Hospital diagnoses (1969-2019) | ICD-8  (1969-1986) | ICD-9  (1987-1995) | ICD-10  (1996-2019) |
| --- | --- | --- | --- |
| Pre-eclampsia | 63703, 63704, 63709, 63799, 63710, 6612 | 6424, 6425, 6426, 6427 | O11, O14, O15 |
| Severe pre-eclampsia | 63704, 63710, 6612 | 6425, 6426 | O14.1, O15 |
| Gestational diabetes | 76110 | 6488 | O24.4 |
| ADPKD | 75310 | 7531A | Q61.2 |
| IA disease | 43000, 43090 | 4300A, 4373 | I60, I67.1, I69.0 |
| Intracerebral hemorrhage | 43100, 43190 | 431 | I61, I69.1 |
| Ischemic stroke | 432-434 | 4330A, 4331A, 4339A, 4340A, 4341A, 4349A | I63, I69.3 |

ADPKD = Autosomal dominant polycystic kidney disease; IA = intracranial aneurysm.
